# The Effect of Bone Marrow-Derived Mesenchymal Stem Cells and Their Conditioned Media Topically Delivered in Fibrin Glue on Chronic Wound Healing in Rats

**DOI:** 10.1155/2015/846062

**Published:** 2015-07-12

**Authors:** Radwa A. Mehanna, Iman Nabil, Noha Attia, Amany A. Bary, Khalid A. Razek, Tamer A. E. Ahmed, Fatma Elsayed

**Affiliations:** ^1^Medical Physiology Department, Faculty of Medicine, Alexandria University, Dr Fahmi Abdel Meguid Street, Mowassat Building, El Shatby, Alexandria 21561, Egypt; ^2^Center of Excellence for Research in Regenerative Medicine and Applications (CERRMA), Faculty of Medicine, Alexandria University, Alexandria 21514, Egypt; ^3^Histology and Cell Biology Department, Faculty of Medicine, Alexandria University, Dr Fahmi Abdel Meguid Street, Mowassat Building, El Shatby, Alexandria 21561, Egypt; ^4^Pathology Department, Faculty of Medicine, Alexandria University, Dr Fahmi Abdel Meguid Street, Mowassat Building, El Shatby, Alexandria 21561, Egypt; ^5^Medical Research Institute, Alexandria University, 71 Victor Emanuel Street, Smouha, Alexandria 21615, Egypt; ^6^Medical Biotechnology Department, Genetic Engineering and Biotechnology Research Institute, City of Scientific Research and Technological Applications, New Borg El-Arab, P.O. Box 21934, Alexandria, Egypt; ^7^Cell Culture Department, Medical Research Institute, Alexandria University, 71 Victor Emanuel Street, Smouha, Alexandria 21615, Egypt

## Abstract

Bone marrow-derived mesenchymal stem cells (BM-MSCs) represent a modern approach for management of chronic skin injuries. In this work, we describe BM-MSCs application versus their conditioned media (CM) when delivered topically admixed with fibrin glue to enhance the healing of chronic excisional wounds in rats. Fifty-two adult male rats were classified into four groups after induction of large-sized full-thickness skin wound: control group (CG), fibrin only group (FG), fibrin + MSCs group (FG + SCs), and fibrin + CM group (FG + CM). Healing wounds were evaluated functionally and microscopically. Eight days after injury, number of CD68+ macrophages infiltrating granulation tissue was considerably higher in the latter two groups. Although—later—none of the groups depicted a substantially different healing rate, the quality of regenerated skin was significantly boosted by the application of either BM-MSCs or their CM both (1) structurally as demonstrated by the obviously increased mean area percent of collagen fibers in Masson's trichrome-stained skin biopsies and (2) functionally as supported by the interestingly improved epidermal barrier as well as dermal tensile strength. Thus, we conclude that topically applied BM-MSCs and their CM—via fibrin vehicle—could effectively improve the quality of healed skin in chronic excisional wounds in rats, albeit without true acceleration of wound closure.

## 1. Introduction

Adult stem cells (ASCs) play an important role in normal homeostasis and repair of the human body. They have been identified within most of the tissues or organs, having multi- or unipotent differentiation potential with a regenerative capacity. These cells ensure normal maintenance of the tissue by efficiently replacing the degenerated ones. Such degeneration-regeneration cycles rejuvenate the tissue and help maintain tissue functions [1].

Bone marrow-derived mesenchymal stem cells (BM-MSCs) represent a heterogeneous population from the non-blood-forming fraction of bone marrow that regulates hematopoietic cell development. In vitro, adult BM-MSCs could differentiate into bone, cartilage, and fat [2]. Furthermore, it has been suggested that they can traverse lineage borders and differentiate into neural cells [3] as well as epithelia of liver, lung, kidney, skin, and the gastrointestinal tract [4]. This issue, however, remains controversial.

Some reports also indicate that MSCs can alter tissue microenvironment by secreting soluble factors and thereby rejuvenate or repair diseased cells and tissues [5]. Such biofactors secreted from MSCs play an important role in various aspects of hematopoiesis and have been named, by some scientists, as “trophic factors” [6]. Thus MSC-induced repair of dysfunctional tissues could be due to their differentiation and/or secretion of such trophic factors.

MSCs have been considered candidates for cell therapy as they can be easily obtained using a simple bone marrow aspiration and can show an extensive capacity for expansion in vitro. So far, MSCs have been used with varying success to improve neurological [7], cardiovascular [8], blood-related [9], and musculoskeletal disorders [10] as well as to treat hard-to-heal cutaneous wounds [11]. Skin has various vital functions, namely, acting as a barrier to foreign pathogens and water loss, also regulating body temperature and supplying sensation [12].

Optimum healing of a skin wound requires an integration of the complex biological and molecular events of cell migration and proliferation, extracellular matrix deposition, angiogenesis, and remodeling [13]. Impairment in such orderly progressing healing process would lead to wound chronicity. Despite having numerous causes, the majority of chronic wounds are associated with diabetes, atherosclerosis, venous/pressure ulcers, vasculitis, and trauma. Given the increasing prevalence of chronic wounds worldwide, besides their marked outcomes on patient morbidity not to mention amputations, it is crucial to consider adequate and effective intervention to treat these debilitating wounds [14].

It has been reported that wounding stimulates BM-MSCs to migrate to the injury site and differentiate into functional skin cells. Nevertheless, the efficiency of MSC migration to the wound is known to be low. Similarly, systemically injected BM-MSCs to treat unhealed wounds would lead to substantial cell loss, hence low therapeutic efficiency [15]. Accordingly, localized cell delivery using biomaterial carriers mimicking the extracellular matrix (ECM) has been reported to improve cell survival and retention [16]. BM-MSCs are candidate cells for such treatment as they release paracrine factors such as erythropoietin (EPO) and granulocyte colony stimulating factor (G-CSF) that enhance the repair/regeneration of nonhematopoietic tissues, including skin wounds [17]. One mechanism through which these paracrine factors influence wound repair is increasing the recruitment of macrophages into the wound thus implying a beneficial effect on wound healing [18].

Regarding the biomaterials, a vast library of them such as collagen, alginate, agarose, hyaluronic acid derivatives, chitosan, and fibrin glue have been used for that purpose. Fibrin is a critical blood component responsible for hemostasis [19]. It was used to promote wound-healing and skin grafting, to provide hemostasis in microvascular surgery and parenchymal injury, and to serve as a matrix for bony fragments in the repair of bone defects [20]. It has been used in regenerative medicine field as a delivery vehicle and scaffolding matrix. In combination with appropriate cell types, fibrin glue has been used in a variety of tissue engineering applications [21].

In this experimental study, Fibrin glue alone, Fibrin glue seeded with MSCs, and Fibrin glue mixed with conditioned media have been applied to induced chronic skin wounds. The aim is to find the most suitable skin substitute among the used models regarding the duration of healing and the restoration of the skin structure and function and also to suggest whether the potential repair was merely due to the seeded MSCs' differentiation or due to their trophic factors secreted into their conditioned media (CM).

Although similar strategies were investigated in this discipline, evaluating its success was mostly behind the functional assessment. Considering the potential of these strategies in the treatment of chronic wound healing, a comprehensive evaluation is needed. Such evaluation should not only include the success of taking of such substitute or the rate by which it promoted healing but also through assessing the function and the durability of such substitutes. Thus, in this study, the function and the mechanical properties of the healed skin were also investigated.

## 2. Materials and Methods

### 2.1. Experimental Animals

Fifty-two adult male Sprague-Dawley rats, weighing 200–250 g, were used at six weeks of age. Each rat had free access to both water and standard rodent soft chow ad libitum. In this study, rats were handled in strict accordance with the guidelines for the care and use of laboratory animals of Faculty of the Medicine, University of Alexandria. The protocol was approved by the institutional review board of animal experiments of Faculty of Medicine, University of Alexandria.

### 2.2. Surgical Procedure and Study Design

Rats were anaesthetized using IM injection of a combination of xylazine HCl and ketamine in doses of 5 and 30 mg/kg body weight, respectively. The dorsal skin was shaved and then disinfected with 10% povidone-iodine before an excisional full-thickness square-shaped skin wound (3.5 × 3.5 cm) was induced using a sterile scalpel (down to the panniculus carnosus). Wound surfaces were covered with semipermeable polyurethane membrane (Tegaderm), and each animal was then housed alone in its cage to avoid any further wound damage. After surgery, all rats were given IM antibiotics (Cefotax 100 mg/mL).

Digital images and wound area measurements were then recorded to visualize and follow up the wound healing process that was estimated based on the remaining wound area. Rats were then randomly divided into four groups (13 rats each, three from each group were sacrificed eight days after wound induction for immunohistochemical study; the rest were left for complete healing). Control group (CG) is negative control where skin injuries were left without treatment. In Fibrin glue treated group (FG) wounds were sprayed with fibrin glue. In Fibrin glue + Stem cells treated group (FG + SCs) wounds were sprayed with MSCs-containing fibrin glue. In Fibrin glue + conditioned media treated group (FG + CM) wounds were sprayed with fibrin glue dissolved in CM (*n* = 10).

### 2.3. Isolation and Culture of BM-MSCs

Rats were euthanized, and femurs and tibias were removed aseptically. A hole was then created in the knee joint end of each bone with a 26-gauge needle, and marrow was flushed with 6 mL complete culture medium (CCM) to isolate bone marrow. CCM was composed of *α*-MEM with addition of 20% FBS, 4% L-glutamine, 2% HEPES buffer, and 2% antibiotics (EuroClone, Wetherby, UK). Flushed bone marrow was centrifuged at 1200 ×g for 20 min. Cell pellets were resuspended in 5 mL CCM and then transferred into T-25 flask and cultured in humidified 5% CO_2_ incubator at 37°C. Medium was changed, and nonadherent cells were removed twice a week. When adherent cells were subconfluent, they were detached with 0.25% trypsin-EDTA solution and reseeded at 2-3 T-25 flasks. MSCs at passage three were used for the current experiments. Follow-up of cultured cells was done using phase contrast inverted microscope (Nikon TSM) equipped with digital camera (DCM 510).

### 2.4. Immunophenotyping of MSCs by Flow Cytometry

To confirm the MSCs' phenotype, we characterized cultured cells for CD44 as one of the surface markers known to be associated with MSCs, in addition to another hematopoietic surface marker (CD45) that is expected to be absent. Cells were detached with 0.25% trypsin-EDTA solution, washed with PBS, and incubated (RT, 30 min, in the dark) with monoclonal PE-conjugated antibodies for CD44 (Abcam, UK) and FITC-conjugated antibodies for CD45 (Abcam, UK). The cells were subsequently washed three times with PBS, resuspended in 500 *µ*L FACS buffer. 10^4^ gated events were acquired and analyzed using a FACS Calibur flow cytometer running CellQuest software (Becton Dickinson, USA).

### 2.5. Preparation of Conditioned Medium (CM)

MSCs at a density of 3 × 10^5^ cells/mL of CCM were seeded in T-25 flasks and subsequently incubated for 48 h; afterwards, CM was harvested and centrifuged at 500 ×g. The supernatant was stored in working aliquots at −20°C till being used.

### 2.6. Preparation and Application of Fibrin Glue

The purchased kits contain two vials of lyophilized human fibrinogen and thrombin (provided by Dr. Hossam M. Fahmy through Cairo Medical Center, Blood Transfusion Center). Each vial was dissolved separately in 1 mL of sterile CCM or CM for FG and FG + CM groups, respectively. The two liquid phases were extruded through a disposable dual chamber applicator and then sprayed through the applicator with an inert gas carrier to form a thin, uniform film of fibrin glue covering the wound. In FG + SCs group, fibrinogen vials were dissolved in 1 mL of MSC suspension (12 × 10^6^ cells/mL CCM) while thrombin vial was dissolved in 1 mL of CCM. The final concentration of fibrinogen and thrombin was 80 mg/mL and 600 units/mL, respectively.

### 2.7. Evaluation of Wound Healing

Skin wounds were photographed using digital camera (Fuji-FinePix S4500) and wounds' surface area was measured for all rats every three days, starting from the day of wound induction (day 0) till complete healing. Duration of wound closure was recorded for statistical comparison. Data were shown as mean ± SD.

### 2.8. Immunohistochemical and Histological Study

Wedge-shaped skin biopsies were obtained by a sharp scalpel (including the granulation tissue formed at the center and the normal surrounding skin at the periphery). Six biopsy specimens were obtained from each group (*n* = 6/group), three of which were obtained 8 days after wound induction, while the other three were after complete wound closure. All specimens were fixed in 10% buffered formalin, embedded in paraffin, and sectioned into 5 *μ*m thick sections.

#### 2.8.1. Immunohistochemistry

For the detection of CD68+ macrophages, sections were deparaffinized and then rehydrated in descending grades of alcohol. The endogenous peroxidase activity was blocked using 3% hydrogen peroxide for 10 min. Following antigen retrieval, anti-CD68 primary antibody (mouse monoclonal Ab, clone KiM6, 1 : 100 dilution) and horseradish peroxide-conjugated secondary antibody (rabbit IgG, 1 : 100 dilution) were used. The bound antibodies were detected using DAB. Positive and negative controls were included in all runs. Finally, slides were counterstained with hematoxylin, mounted, and cover slipped. The antibodies and the detection system were provided by Lab Vision Corporation (Neo Markers, Fremont, USA). Microscopic evaluation of the macrophage infiltration was done semiquantitatively using bright-field light microscopy coupled with digital camera. Ten different random high power fields (HPF) were examined in each specimen and the infiltrating macrophages were counted. Data were presented as mean ± SD (*n* = 3/group).

#### 2.8.2. Masson's Trichrome Staining for Collagen Fibers

After deparaffinization and rehydration of sections, they were stained with Masson's trichrome to demonstrate collagen fibers. Micrographs were captured from each sample at 100x magnification. By using the threshold color tool in the NIH Fiji^©^program, the areas stained in green were selected and in that way we could indirectly calculate the area fraction of collagen in the dermis. As previously described by Pereira and coworkers [22], the ratio of green pixels above the threshold to total pixels in the image is used to calculate the percentage of collagen in each sample, and thus we could compare between groups. Data were reported as mean ± SD (*n* = 3/group).

### 2.9. Functional Assessment of Healed Skin

These parameters were measured after complete wound closure to determine and compare the restoration of skin functional integrity.

#### 2.9.1. Skin Hydration

Hydration is defined as the water content of the Stratum Corneum (SC). Skin hydration was assessed by EnviroDerm Services, Corneometer CM825 (Dermal measurement system EDS12, UK). This probe uses fringing field capacitance sensors to measure the dielectric constant of the skin that normally changes with water content of the SC. These changes are converted into arbitrary units of hydration. Values from 1 to 4 are interpreted as dry skin (1 being the least moist), values from 5 to 8 are for normally hydrated skin, and finally values from 9 to 12 are interpreted as excessively hydrated skin (12 being the most moist). Data were shown as mean ± SD (*n* = 10/group) [23].

#### 2.9.2. Transepidermal Water Loss (TEWL)

Measurement of TEWL was used for studying the water barrier function of the epidermis. The more efficient the skin waterproof barrier character, the higher the water content and the lower the TEWL. TEWL was measured using EnviroDerm Services Tewameter (Dermal measurement system EDS12, UK). The principle of the device was described in detail by Nilsson [24]. A boundary layer develops around the skin in which water vapor gradient exists between the skin surface and ambient air. The sensors of the EDS12 probes (pairs of hygrosensors and thermosensors), mounted in the open chamber of the probes, determine the water vapor pressure gradient of this boundary layer in order to quantify the diffusion of water through the skin. Its value was expressed in g/h/m^2^ [mass of water vapor (g) per area (m^2^) and time (h)].

The values of TEWL recorded were interpreted according to the used equipment guide. Where the values from 0 to 12 indicate healthy skin barrier function (0 being the best functioning barrier), values from 13 to 16 indicate strained skin barrier function while values from 17 to 20 indicate critical skin barrier function (20 being the most damaged barrier).

During all the measurements, either probe was hand held using an insulating glove, thus avoiding heating of the probe. Displayed values were read. All measurements were performed inside an incubator to avoid convection of air. Data were shown as mean ± SD (*n* = 10/group).

#### 2.9.3. Tensile Strength Assessment

Skin is subject to tensile stress. Confounding factors such as the thickness of the skin, the surface area being tested, and the type of forces applied were all standardized. The force meter apparatus (Mark-10, Model BG500-USA) was used to characterize the tensile strength of the healed skin of all groups. A rectangular sample of the healed skin, about 1.0 cm wide, was fixed to the force gauge and subjected to tension at a speed of 1 mm/minute until failure. Assessment was carried out at room temperature. The tensile strength and extension ratio were calculated from the force and displacement values obtained from the experiment. The tensile strength in kg/mm^2^ was calculated through dividing the breaking strength (kg) by the wound surface area (mm^2^) [25]. Data were shown as mean ± SD (*n* = 7/group).

### 2.10. Statistical Analysis

Data were fed to the computer and analyzed using IBM SPSS software package version 20.0. Quantitative data were described using mean and standard deviation. The distributions of quantitative variables were tested for normality using Kolmogorov-Smirnov test, Shapiro-Wilk test and D'Agstino test. Also, Histogram and QQ plot were used for vision test. Data were normally distributed, and, thus, comparison between different studied groups was analyzed using *F*-test (ANOVA) and post hoc test (LSD) for pair-wise comparisons. Significance of the obtained results was judged at *p* ≤ 0.05.

## 3. Results

### 3.1. Morphologic and Immunophenotypic Characterization of MSCs

**Figure 1:**
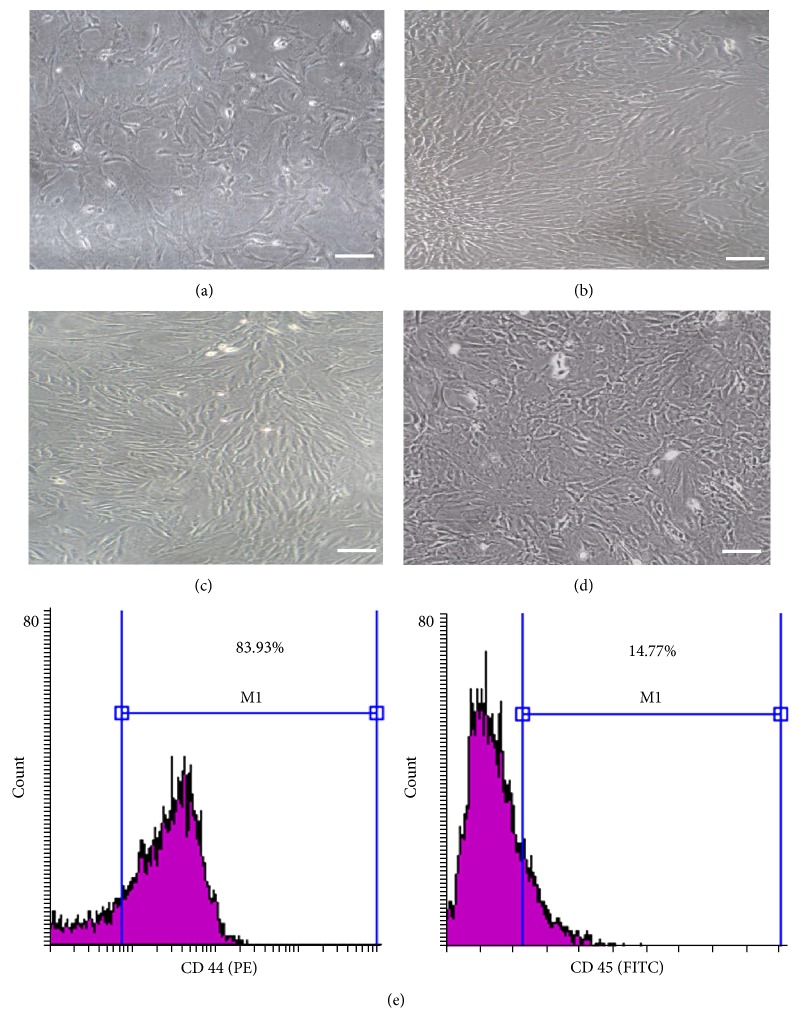
Morphological and phenotypic characteristics of BM-MSCs. The cells isolated from rat bone marrow exhibited spindle shaped fibroblast-like morphology. (a) Primary culture, passage zero (P0), (b) P1, (c) P2, and (d) P3. (Phase contrast microscope, scale bar = 100 *µ*m.) (e) A representative flow cytometric analysis of cell-surface markers of BM-MSCs at P3. Cells were labeled with antibodies against MSC marker (CD44-PE) and hematopoietic antigen (CD45-FITC); M1 is the % of cells positive for both markers.

Shortly after culture, fusiform or polygonal cells showed multiple small cytoplasmic projections adhering to the tissue culture plastic. Cells continue to proliferate and became confluent within two weeks, in general. We first focused on the morphology of the cultured cells at different passages. This is shown in Figures 1(a)–1(d) where cells depicted heterogeneous morphology, exhibiting large, flattened, and slim spindle shapes. Cultures of P0–P3 were photographed when they were almost semiconfluent, denoting that cellular morphology and proliferative potential were maintained in the passages adopted in this study.

FACS analysis demonstrated that 83.93% of the cultured cells expressed the mesenchymal CD44 marker, albeit 85.23% depicted the absence of the hematopoietic CD45 marker (Figure 1(e)).

### 3.2. Follow-Up of Wound Healing

**Figure 2:**
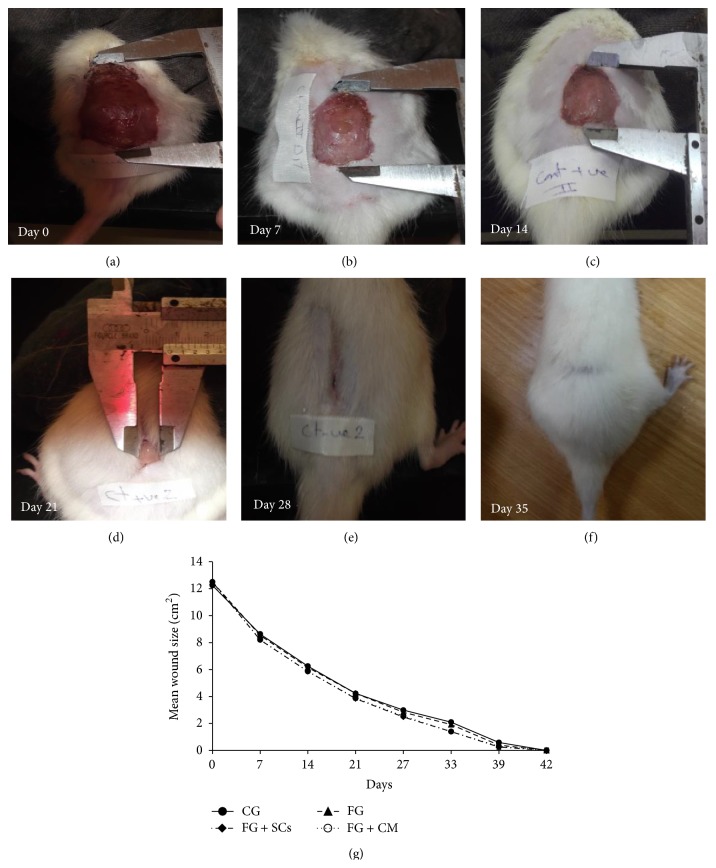
Model of progression of wound healing over time and wound measurement. (a)–(f) The shown graduated instrument measured the wound length and width. (a) Day 0. (b) Day 7. (c) Day14. (d) Day 21. (e) Day 28. (f) Day 35 complete healing. (g) Line graph of wound healing progression over time. Vertical error bars represented the standard deviation of the reported mean values (*n* = 10).

Measurement of wounds' size was gradually decreasing over time (Figure 2). Wounds' size in FG + SCs and FG + CM groups showed significant decrease in comparison to the CG and FG during most of healing duration (till day 27) after which they were mostly indifferent till the point of complete healing. On the other hand, there was no difference in wounds' size neither between the CG and FG nor between FG + SCs and FG + CM groups along the whole wound healing duration (supplementary table in Supplementary Material available online at http://dx.doi.org/10.1155/2015/846062). However our data (Table 1) showed no significant difference between groups regarding the duration of wound closure (*p* = 0.426).

### 3.3. Immunohistochemical and Histological Study

#### 3.3.1. Immunohistochemistry

**Figure 3:**
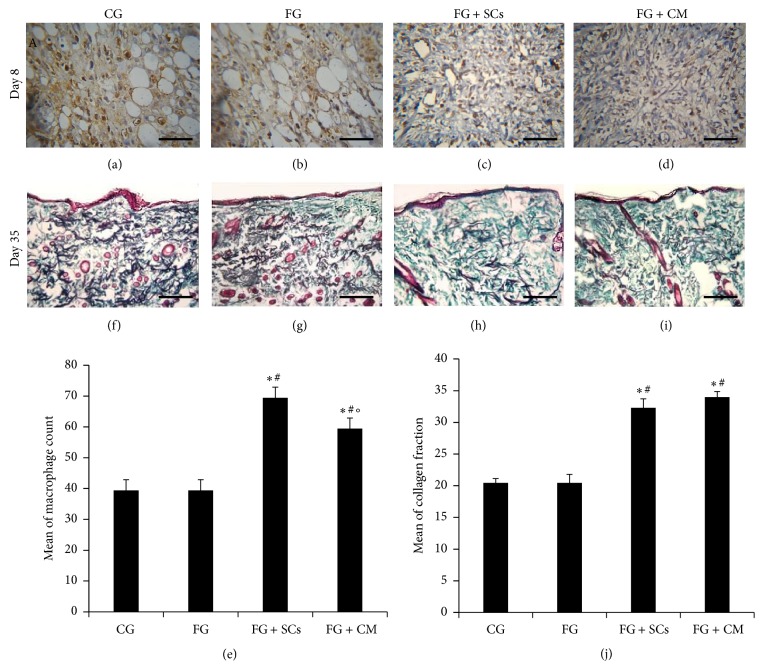
(a)–(d) Immunohistochemistry of granulation tissue 8 days after injury showing variable degrees of immunoreactivity in CD68+ macrophages (brown color). Each figure is a hematoxylin-counterstained representative section (Scale bar = 50 *µ*m). (e) Macrophage count/HPF. (f)–(i) Photomicrographs of skin biopsies 35 days after injury depicting variable amounts and arrangement of collagen fibers in the dermis. Each figure is a Masson's trichrome-stained representative section (scale bar = 200 *µ*m). (j) Histomorphometric quantification of mean collagen fraction. In (e) and (j) *F*-test (ANOVA) test was used to analyze data presented as mean ± SD; “*∗*” is significant versus CG and “#” is significant versus FG group [*p* < 0.001], while “°” is significant versus FG + CM [*p* < 0.05] (*n* = 3).

Examination of hematoxylin-counter stained sections revealed the central part of the wound with ulcerated skin surface, the dermis and subcutaneous tissue show a granulation tissue formed of small newly formed capillary-sized blood vessels lined by plump endothelial cells and proliferating fibroblasts, and the intervening stroma is edematous and shows variable numbers CD68+ macrophages admixed with other inflammatory cellular infiltrate (Figures 3(a)–3(d)). The mean number of macrophages/HPF was 39.44 ± 3.47 in both CG and FG, 69.44 ± 3.47 in FG + SCs and 59.44 ± 3.47 in FG + CM. As shown in Figure 3(e), no difference was detected between numbers of macrophages in the first two groups. However, in FG + SCs and FG + CM groups, their count was significantly higher than FG and CG groups (*p* < 0.01). The samples of FG + SCs group were more infiltrated by macrophages than FG + CM group (*p* < 0.05).

#### 3.3.2. Masson's Trichrome Stain for Collagen Fibers

As seen in Figures 3(f)–3(i), both CG and FG groups depicted deposition of homogenous amorphous intercellular substance and few collagen fibers aligned horizontally in one direction. On the other hand, examination of skin samples in FG + SCs and FG + CM treated groups revealed dense arrangement of thick collagen fibers in different directions in a network-like manner. These findings were confirmed by the data illustrated in Figure 3(j). In CG and FG, the percentage of collagen was 20.44 ± 0.69 and 19.10 ± 1.36, respectively. Nevertheless, the percentage of collagen was evidently increased in FG + SCs group to be 33.99 ± 0.87 quite close to 32.29 ± 1.42 percent in FG + CM group.

### 3.4. Functional Assessment of Healed Skin

#### 3.4.1. Hydration

The mean hydration value of FG + SCs group was 5.0 ± 0.82, which lies in the range of the normally hydrated skin. While that of FG + CM was 4.70 ± 0.67 and thus lies in the slightly dry skin range. On the other hand, the hydration mean values in CG and FG were 1.50 ± 0.85 and 1.60 ± 0.70, respectively, being within the range values of extremely to very dry skin.

**Figure 4:**
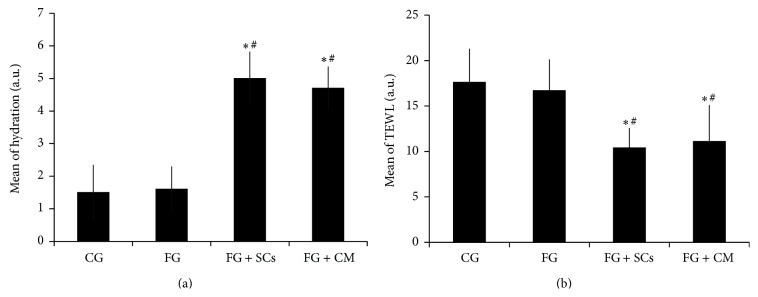
Comparison between the different studied groups according to hydration (a) and TEWL (b). One-way ANOVA test was used to analyze the data presented as mean ± SD; “*∗*” is significant versus CG; “#” is significant versus FG group [*p* < 0.001] (*n* = 7).

Data shown in Figure 4(a) attests that hydration values were significantly higher in FG + SCs and FG + CM groups in comparison to the CG and the FG groups (*p* < 0.001). However, there was no significant difference in its value neither between FG + SCs and FG + CM groups nor between FG and CG groups.

#### 3.4.2. TEWL

The mean TEWL values of CG and FG groups were 17.60 ± 3.69 and 16.70 ± 3.43, respectively, being in the range of critical skin barrier function. Their values were significantly higher than FG + SCs and FG + CM groups that had values of 10.40 ± 2.17 and 11.10 ± 4.01, respectively, and thus lie in the range of healthy skin barrier function. However, data demonstrated no significant difference between the two later groups (Figure 4(b)).

#### 3.4.3. Tensile Strength and Extension Ratio

The tensile strength and extension ratios were significantly higher in FG + SCs and FG + CM in comparison with CG and FG groups. Nevertheless, there was no significant difference neither between FG + SCs and FG + CM groups nor between FG and CG groups regarding both parameters (Table 1).

## 4. Discussion

Chronic wounds are generally marked by their delayed healing. However, when it comes to the specifics of how much time is necessary before a wound is classified as “chronic” and the factors affecting its chronicity, definitions are still under discussion. In the present study we adopted the definition of Lazarus and Stacey for chronic wounds as “unhealed wound that have been present for more than one month” [26]. Chronicity could be due to the wound size, superadded infection/foreign body, or perturbed immune response. Now, it is obvious that chronic wounds are multifactorial in etiology and thus the lack of success of single-agent growth factor therapy is not surprising [27].

Developing an animal model depicting the same complexity of human chronic wounds is so far an unattainable goal [28]. In this work, we induced chronic wound in rats through a square-shaped full-thickness excisional wound model (3.5 cm × 3.5 cm) that was sufficiently large to remain unhealed after one month, thus recognized as chronic. When smaller wound dimensions or rounded wound shapes were probed, healing occurred in less than one month. However, larger wound sizes had compromised animals' survival (data not shown). Knowing that wound contraction is one of the most significant limitations to using loose-skinned animals to model human wounds, we extended the excision to include the layer of panniculus carnosus muscle. This method was suggested by Karypidis et al. [29] to alleviate skin contraction. Thus, data derived from the wound closure are more representative of healing by secondary intention (reepithelialization and granulation tissue (GT) formation).

The restoration of tissue integrity is the result of the interaction of multiple factors including blood platelets and cells, such as neutrophils, macrophages, fibroblasts, endothelial cells, and keratinocytes as well as extracellular matrix components [30, 31]. The coordination and the integration between the previously mentioned factors are regulated by stem cells [32, 33].

Bone marrow-derived stem cells have been reported to secrete myriad factors acting in a paracrine manner to promote angiogenesis, alter cell migration, and inhibit apoptosis [34, 35]. Such factors could be identified and obtained from their conditioned media [36]. Following skin wound injury, MSCs secrete several paracrine factors including vascular endothelial growth factor (VEGF), transforming growth factor beta 1 (TGF-*β*1), platelet derived growth factors (PDGF), insulin-like growth factors (IGF), interleukin 6–8, and others [37].

Evidence for the role of some of these factors was described by Chen et al., 2008 [18] in mice wound healing model, in particular the role of IGF-1 as shown by the extreme elevation of its gene expression in BM-MSC conditioned medium. It acts by dampening the local inflammatory response and promoting reepithelialization [38]. Also, as reviewed by Penn et al., 2012 [39], TGF-*β* is involved in inflammation, fibroblast proliferation, collagen synthesis, and deposition/remodeling of new extracellular matrix. Furthermore, VEGF, PDGF, and IGF-1 were proved to be chemotactic to endothelial cells and enhance their proliferation and thus promote new blood vessels formation [18]. Being devoid of any cell type, conditioned media would be a safe means for clinical trial setting as it eliminates the concern of potential stem cell transformation. Also, easy preparation, long term storage, and avoidance of potential limitations of cell-based treatments shed valuable light on its potential clinical practicality.

Optimal wound healing involves several events, starting with hemostasis, the phase that begins immediately after wounding with vascular constriction and fibrin clot formation. Fibrin sealants have been approved for hemostasis in the USA and Europe and are occasionally used to promote wound healing. However, inconsistency exists in the literature regarding the benefit of these preparations in other events of the healing process [40]. The usage of fibrin glue per se as a treatment delivery system, for the cultured cells and conditioned media, did not influence the assessed parameters in the present work. Results showed no difference between the control untreated group and the fibrin glue applied one.

This was in accordance with other studies [41, 42], claiming that the fibrin glue is only a vehicle rather than a healing promoter. In their published works Mandez et al., [41] and Rehder et al. [42] had concluded that the mere application of fibrin glue did not improve wound healing unless being combined with mesenchymal stromal cells and keratinocytes, respectively.

On contrary, in their in vitro model of wound reepithelialization, Geer and colleagues [43] showed that fibrin affects keratinocyte activation and increases the consistency of the healing response. An interpretation to such discrepancy may largely depend on the composition of fibrin biomatrices that might considerably control its effect on wound healing [44]. Additionally, the efficacy of fibrin sealant depends greatly on the surgical situation it is used in [40].

Once bleeding is controlled, inflammatory cells, including macrophages, are recruited to the wound area to promote the inflammatory phase [45, 46].

Macrophages play multiple roles in wound healing. In the early wound, macrophages release cytokines that promote the inflammatory response by recruiting and activating additional leukocytes. In parallel, they stimulate keratinocytes, fibroblasts, and angiogenesis to promote tissue regeneration [47, 48]. They are also responsible for clearing of apoptotic cells, thus paving the way for the resolution of inflammation. In this way, macrophages promote the transition to the proliferative phase of healing.

In our study, data showed that wounds sprayed with fibrin glue admixed with BM-MSCs or their conditioned medium had significantly increased numbers of macrophages when compared to FG and control groups. According to the previously mentioned role of macrophages in healing, we can postulate that their increased number in the two former groups lead to better clearing of apoptotic cells and thus improving the healed skin structure and function. Such finding is consistent with data published by Chen and colleagues [18] depicting that CM of BM-MSCs were strongly chemoattractive to monocytes. Further supportive findings of Seebach et al. [49] attested that cell-free fibrin implants were not invaded by chemoattracted immune cells, Along with Hong and colleagues [50] who emphasized that macrophage infiltration enhances the granulation tissue, thus promoting proper healing, though no significant enhancement of the reepithelialization of wounded skin was seen.

In our study the use of FG + SCs and FG + CM strategies did not accelerate the rate of wound reepithelialization, similar to the recently published results of Hong et al. [50]. Nevertheless, there has been a significant improvement regarding the functional and histological quality of healed skin, assessed through its epidermal barrier function as well as dermal mechanical property.

On the other hand, Wu et al. [51] reported that topical application of MSCs accelerates chronic wound healing via secretion of varieties of cytokines and growth factors and/or the ability of the stem cells to differentiate into fibroblasts and keratinocytes responsible for synthesis of extracellular matrix constituents. Additionally, Chen et al. [18] showed that CM of BM-MSCs containing high levels of growth factors and chemokines accelerates wound healing in mice, implying a critical role of paracrine factors in MSC-mediated enhanced wound healing. A 6 mm full-thickness excisional skin wound model was induced in both studies, and such far smaller wound size adopted could be behind the discrepancy between their results and ours concerning the healing rate.

The stratum corneum forms a barrier against diffusion of water through the epidermis; hydration measures its water binding capacity while the measurement of TEWL provides information concerning the integrity of the epidermis. Lower values of TEWL and higher values of SC hydration are, in general, characteristic features of a healthy, intact skin barrier [52].

Our results revealed that both skin barrier function and hydration were only restored in both MSCs and CM treated groups with no true difference between them. Likewise, Lee et al. [53] had shown that the topical application of CM of ADSC in the treatment of atopic dermatitis led to improvement of epidermal permeability barrier and keratinocyte differentiation. TEWL and skin hydration were also used to assess the skin functions in other studies, since Loannovich et al. [54] concluded TEWL as an objective and noninvasive method of measuring wound healing and assessing epithelial repair.

Another important aspect of the healed skin quality is the restoration of its dermal mechanical properties that depend, to a large degree, on the specific arrangement of collagen fibers. The skin's tensile strength is able to investigate such properties and is believed to have such a profound effect on wound healing. Our results showed a significantly higher tensile strength as well as extension ratios in MSCs and CM treated groups when compared to CG and FG groups. Gallagher et al. [55] had studied the mechanical properties of the skin using the tensile strength tests and concluded that stress strain data and physical properties can be used to facilitate the determination of material parameters for structurally based constitutive models for skin.

The improved tensile strength and extension ability of the healed skin treated with MSCs or CM were correlated to the histological structure of this skin as shown in the current study. Despite synchronous wound closure of CG and FG groups, collagen fibers were still relatively thin and oriented parallel to the skin indicating improper wound maturation. On the opposite side, FG + SCs and FG + CM groups had thicker collagen fibers that were arranged in a network-like manner mimicking normal skin. These findings might be attributed to the increased number of macrophages in both stem cells and CM treated groups. Such macrophages are believed to control the synthesis of collagen through their secreted growth factors [56]. They are also reported to be responsible for better remodeling of dermal fibrous architecture, through the resorption of the earlier deposited thin collagen fibrils (type III) that were later replaced with thicker fibrils (type I) aligned with stress lines indicating wound maturity [57].

In addition to the fact that the results of this work can serve as an experimental model for further research using various factors by which the process of skin wound healing can be influenced the study also has a clinical importance as it demonstrated almost the same effect of MSCs-based and MSCs-free conditioned media therapies on the physiological, mechanical, and histological properties of healed skin. This finding could indirectly answer the question of whether the regenerative effect of stem cells is due to their differentiation or due to the effect of the various growth factors they secrete. Also, based on these findings, the development of cell-free products that can be applied to wounds and improve the quality of healed skin would be a promising issue in this discipline leaving back the hazards and limitations of cell-based treatments.

Nonetheless, trying to probe possible modalities to treat chronic wounds, the present data still have certain limitations; the issue of chronic wound model requires further optimization before considering the potential implications of these findings on humans [58]. Notwithstanding several scientific and ethical concerns about the use of FBS in stem cell culture, to date, its use has not been totally replaced by chemically defined or even serum-free culture media. This is mainly because of the higher cost, limited availability, need for different cell-tailored recipes, and most importantly their unsatisfactory support for cell proliferation. Likewise, although our provisional results display the positive influence of MSCs and/or their conditioned media on wound healing, thorough investigations are still needed to reveal the molecular bases behind such observations.

## 5. Conclusion

Topically applied BM-MSCs or their CM, via fibrin vehicle, effectively improved the quality of healed skin in chronic excisional wounds in rats, albeit without true acceleration of wound closure.

## Supplementary Material

Line graph of wound healing progression over time where wounds' size in FG + SCs and FG + CM groups showed significant decrease in comparison to the CG and FG during most of healing duration (till day 27) after which they were mostly indifferent till the point of complete healing. On the other hand, there was no difference in wounds' size neither between the CG and FG nor between FG + SCs and FG + CM groups along the whole wound healing duration.
The mean wound sizes (cm^2^) in relation to time (days) is also shown in the table where significant difference in wound size was shown among different groups from one hand and among the same group with progression of time from the other hand.

## Figures and Tables

**Table 1:** Comparison between the different studied groups according to tensile strength, extension ratio, and healing duration.

	CG	FG	FG + SCs	FG + CM	*p*
Tensile strength (Kg/mm^2^)	0.015 ± 0.001	0.013 ± 0.003	0.023^ab^ ± 0.007	0.020^ab^ ± 0.002	<0.001^*∗*^
Extension ratio (mm)	1.63 ± 0.21	1.65 ± 0.14	2.13^ab^ ± 0.27	1.99^ab^ ± 0.21	<0.001^*∗*^
Healing (days)	36.90 ± 4.31	36.30 ± 4.06	34.40 ± 3.66	34.60 ± 4.03	0.426

Data are presented as mean ± SD (tensile strength and extension ratio: *n* = 7/group, healing: *n* = 10/group). One-way ANOVA was conducted and post hoc test for pair comparison. ^*∗*^Results are significant at *p* < 0.001. CG: control group, FG: wound sprayed with fibrin glue, FG + SCs: wound sprayed with MSCs-loaded fibrin glue, and FG + CM: wound sprayed with fibrin glue dissolved in conditioned media. ^a^Significant with CG and ^b^significant with FG.
