# Supplementary material for: The Effect of Bone Marrow-Derived Mesenchymal Stem Cells and Their Conditioned Media Topically Delivered in Fibrin Glue on Chronic Wound Healing in Rats
Source: Biomed Res Int. 2015 Jul 12;2015:846062. doi: 10.1155/2015/846062 (PMC4508387; doi:10.1155/2015/846062)
Supplement: Supplementary file 1 — Line graph of wound healing progression over time where wounds' size in FG + SCs and FG + CM groups showed significant decrease in comparison to the CG and FG during most of healing duration (till day 27) after which they were mostly indifferent till the point of complete healing. On the other hand, there was no difference in wounds' size neither between the CG and FG nor between FG + SCs and FG + CM groups along the whole wound healing duration. The mean wound sizes (cm2) in relation to time (days) is also shown in the table where significant difference in wound size was shown among different groups from one hand and among the same group with progression of time from the other hand. [file 846062.f1.pdf]

Supplementary data

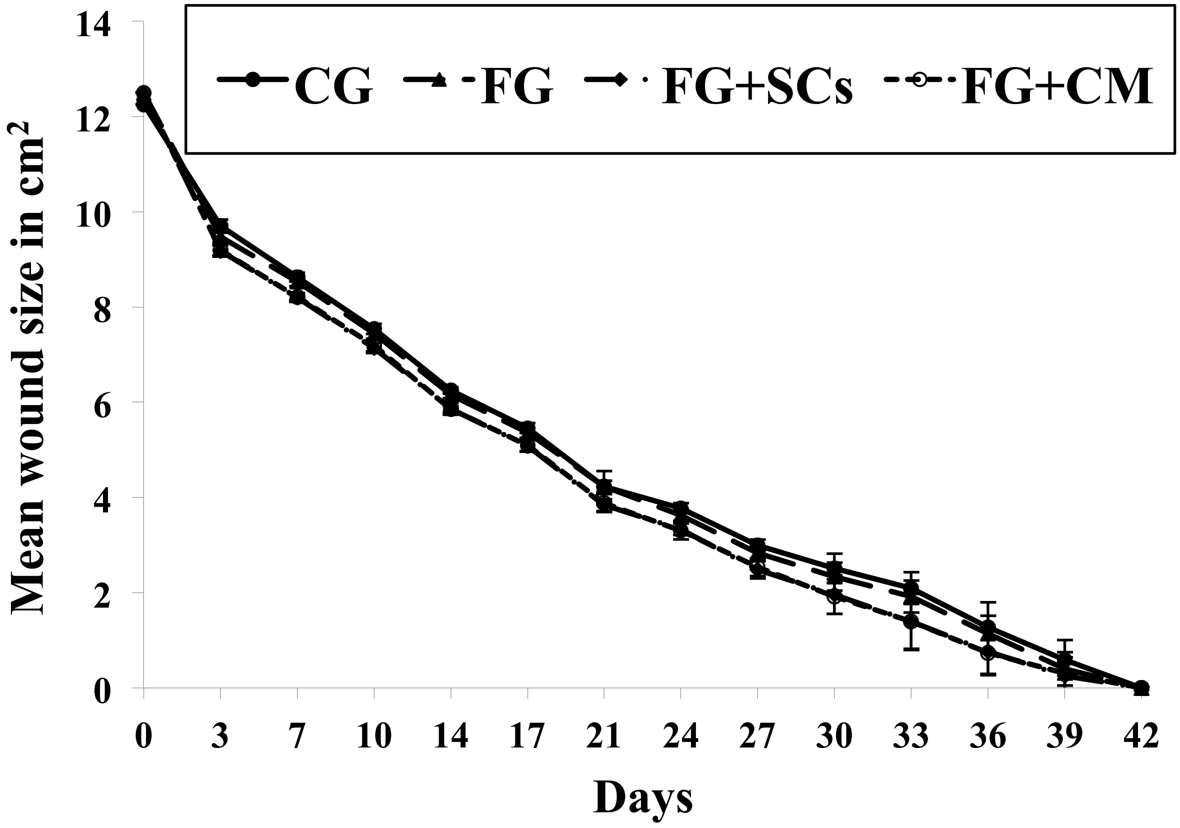


Supplementary Figure: Line graph of wound healing progression over time. Vertical error bars represented the standard deviation of the reported mean values. (n = 10)

**Supplementary Table: Comparison between the different studied groups according to wound size ($cm^2$) in relation to time (days) ($n = 10$)**

| | CG | FG | FG+SCs | FG+CM | p |
|---|---|---|---|---|---|
| **Day 0** | $12.25 \pm 0.0$ | $12.50 \pm 0.0$ | $12.50 \pm 0.0$ | $12.50 \pm 0.0$ | - |
| **Day 3** | $9.70^{\#} \pm 0.14$ | $9.47^{a\#} \pm 0.12$ | $9.18^{ab\#} \pm 0.12$ | $9.19^{ab\#} \pm 0.11$ | $<0.001^{*}$ |
| **Day 7** | $8.63^{\#} \pm 0.09$ | $8.53^{\#} \pm 0.09$ | $8.20^{ab\#} \pm 0.09$ | $8.21^{ab\#} \pm 0.07$ | $<0.001^{*}$ |
| **Day 10** | $7.54 \pm 0.11$ | $7.44^{\#} \pm 0.11$ | $7.14^{ab\#} \pm 0.11$ | $7.19^{ab\#} \pm 0.12$ | $<0.001^{*}$ |
| **Day 14** | $6.25^{\#} \pm 0.07$ | $6.15^{\#} \pm 0.07$ | $5.86^{ab\#} \pm 0.07$ | $5.86^{ab\#} \pm 0.13$ | $<0.001^{*}$ |
| **Day 17** | $5.46 \pm 0.11$ | $5.36^{\#} \pm 0.11$ | $5.08^{ab\#} \pm 0.11$ | $5.09^{ab\#} \pm 0.13$ | $<0.001^{*}$ |
| **Day 21** | $4.23^{\#} \pm 0.12$ | $4.23^{\#} \pm 0.33$ | $3.84^{ab\#} \pm 0.13$ | $3.88^{ab\#} \pm 0.19$ | $<0.001^{*}$ |
| **Day 24** | $3.77^{\#} \pm 0.11$ | $3.63 \pm 0.13$ | $3.33^{ab\#} \pm 0.13$ | $3.31^{ab\#} \pm 0.19$ | $<0.001^{*}$ |
| **Day 27** | $2.99^{\#} \pm 0.12$ | $2.83 \pm 0.13$ | $2.48^{ab\#} \pm 0.18$ | $2.54^{ab\#} \pm 0.19$ | $<0.001^{*}$ |
| **Day 30** | $2.51 \pm 0.30$ | $2.34^{\#} \pm 0.30$ | $1.96^{a} \pm 0.40$ | $1.92^{a\#} \pm 0.37$ | $0.001^{*}$ |
| **Day 33** | $2.10^{\#} \pm 0.34$ | $1.92^{\#} \pm 0.34$ | $1.40^{a\#} \pm 0.60$ | $1.40^{a} \pm 0.58$ | $0.003^{*}$ |
| **Day 36** | $1.28 \pm 0.52$ | $1.14^{\#} \pm 0.38$ | $0.76^{\#} \pm 0.46$ | $0.74^{\#} \pm 0.47$ | $0.026^{*}$ |
| **Day 39** | $0.59 \pm 0.41$ | $0.40^{\#} \pm 0.35$ | $0.26^{\#} \pm 0.28$ | $0.31 \pm 0.33$ | 0.174 |
| **Day 42** | $0.0 \pm 0.0$ | $0.0 \pm 0.0$ | $0.0 \pm 0.0$ | $0.0 \pm 0.0$ | - |

p: p value for F test (ANOVA), Sig. bet. groups was done usingPost Hoc Test (Scheffe)
a: significant with CG
b: significant with FG
#: Significant with the previous wound size in the same group
#*: Statistically significant at $p \le 0.05$
